# Perceived teacher support in ideological and political education and life satisfaction among Chinese university students: an indirect association through presence of meaning in life

**DOI:** 10.3389/fpsyg.2026.1978518

**Published:** 2026-10-07

**Authors:** Mingchao Ma, Wei Liu

**Affiliations:** 1 School of Marxism, Hezhou University, Hezhou, China; 2 Graduate School, José Rizal University, Mandaluyong, Philippines

**Keywords:** Chinese university students, higher education, ideological and political education, life satisfaction, perceived teacher support, presence of meaning in life

## Abstract

**Background:**

Supportive teacher–student relationships are increasingly recognized as relevant to university students’ psychological functioning, but their role within ideological and political education (IPE) remains comparatively underexamined. This study examined the associations among perceived teacher support in IPE, presence of meaning in life, and life satisfaction among Chinese university students, with particular attention to the indirect association through presence of meaning.

**Methods:**

A cross-sectional survey was conducted with 365 undergraduate students recruited from three higher education institutions in Guangxi, China. Perceived teacher support was assessed using a contextualized version of the Perceived Teacher Support Scale across instrumental, emotional, informational, and appraisal support, while presence of meaning and life satisfaction were measured using established scales. Confirmatory factor analysis and structural equation modeling were used to evaluate the measurement and hypothesized structural models. Indirect associations were examined using 5,000 bias-corrected bootstrap resamples.

**Results:**

The hypothesized structural model showed good fit, χ^2^(553) = 810.93, CFI = 0.959, TLI = 0.956, RMSEA = 0.036, and SRMR = 0.053. Perceived teacher support was positively associated with presence of meaning in life (*β* = 0.305, 95% bias-corrected bootstrap CI [0.181, 0.423]) and life satisfaction (*β* = 0.191, 95% BC bootstrap CI [0.076, 0.306]). Presence of meaning in life was also positively associated with life satisfaction (*β* = 0.421, 95% BC bootstrap CI [0.299, 0.534]). A significant indirect association between perceived teacher support and life satisfaction through presence of meaning in life was observed (*β* = 0.128, 95% BC bootstrap CI [0.073, 0.199]). The model explained 9.3% of the variance in presence of meaning in life and 26.2% of the variance in life satisfaction.

**Conclusion:**

Perceived teacher support within university IPE was associated with both students’ presence of meaning and life satisfaction, and part of the association between perceived teacher support and life satisfaction was statistically represented through presence of meaning within the specified model. The findings extend teacher-support research into a value-oriented educational context and highlight the relevance of students’ perceptions of supportive teacher–student relationships to presence of meaning and broader evaluations of life.

## Introduction

Life satisfaction is an important indicator of university students’ positive psychological functioning and refers to individuals’ global cognitive evaluation of the quality of their lives (Diener et al., 1985; Diener et al., 1999). University students encounter multiple academic, interpersonal, and developmental demands, making their educational and social environments relevant to how they evaluate their lives (Campbell et al., 2022; Khatri et al., 2024). Academic demands and limited educational resources have been associated with poorer academic well-being and life satisfaction, whereas growth opportunities, institutional support, and supportive interpersonal relationships may function as beneficial resources (Scheepers et al., 2024; Versteeg and Kappe, 2021). Social support has likewise been associated with lower stress and more favorable mental-health and well-being outcomes among university students (McLean et al., 2023; Li et al., 2025). Similar associations between social and psychological resources and positive psychological functioning have been reported among Chinese university students (Ye et al., 2021; Xu and Choi, 2023; Ding et al., 2024). These findings provide a basis for examining how supportive experiences within university education are associated with students’ broader evaluations of their lives.

Teachers represent one potentially important source of support within higher education. Perceived teacher support has been associated with students’ motivation, psychological need satisfaction, and positive psychological functioning (Pineda et al., 2025). Evidence from Chinese university students similarly indicates that supportive teacher–student relationships are associated with psychological well-being and positive educational experiences through more proximal motivational and psychological resources (Liu and Zhao, 2026; Kaur et al., 2024; Wang et al., 2023). These findings provide a basis for conceptualizing perceived teacher support as a contextual interpersonal resource within higher education.

Ideological and political education (IPE) provides a relevant context in which to examine perceived teacher support because university IPE courses in China combine academic instruction with value-oriented themes concerning personal development, social responsibility, and relationships between individuals and society. Recent research has linked teacher–student relationships in IPE with students’ motivation and academic emotions (Wang et al., 2024), while teacher support and influence have also been associated with students’ emotional engagement in university ideological and political theory courses (Wang, 2026). Other studies have examined broader psychological outcomes within IPE, including psychological nurturing, psychological capital, and subjective well-being (Xue et al., 2024; Zhao and Yang, 2026). However, participation in IPE itself cannot be assumed to produce uniformly positive psychological outcomes. Students may differ substantially in the extent to which they perceive their teachers as responsive, encouraging, informative, and constructive. The present study therefore focuses on perceived teacher support within IPE rather than exposure to IPE itself.

Presence of meaning in life may be particularly relevant to understanding how supportive educational experiences correspond with life satisfaction. It refers to the extent to which individuals experience their lives as purposeful, coherent, and significant rather than actively searching for meaning (Steger et al., 2006; Martela and Steger, 2016). Research among university students has associated interpersonal and psychological resources with a stronger sense of meaning in life (Lyu, 2024; Cai and Zhan, 2025). Presence of meaning, in turn, has been consistently associated with more favorable evaluations of life and psychological functioning (Zhang et al., 2025; Ortega-Gómez et al., 2025). Presence of meaning therefore represents a plausible proximal psychological resource linking supportive interpersonal experiences with broader evaluations of life.

Despite these converging lines of research, teacher support, meaning in life, life satisfaction, and ideological and political education have largely been examined in separate strands of literature. Existing studies have examined teacher support in relation to outcomes such as motivation, engagement, need satisfaction, and psychological functioning, while meaning-in-life research has considered factors including social support, connectedness, stress, hope, and well-being. In parallel, IPE research has increasingly considered emotional engagement, psychological capital, well-being, and teacher influence, but has rarely examined perceived teacher support as a multidimensional interpersonal resource in relation to both meaning in life and life satisfaction. Accordingly, this study examines the direct and indirect associations among perceived teacher support in ideological and political education, presence of meaning in life, and life satisfaction among Chinese university students within a theoretically specified model. By doing so, it shifts attention from participation in ideological and political education toward the interpersonal support students perceive within that context and examines presence of meaning as a proximal psychological resource that may be statistically relevant to the association between supportive educational experiences and students’ broader evaluations of their lives.

## Literature review and hypotheses

### Perceived teacher support in ideological and political education and presence of meaning in life

The proposed model adopts a resource-oriented conceptual perspective that distinguishes between contextual interpersonal resources and more proximal psychological resources. Perceived teacher support represents a contextual resource embedded in students’ educational relationships and refers to students’ perceptions that teachers provide instrumental assistance, emotional encouragement, useful information, and constructive feedback. Such support may be psychologically relevant not merely because it facilitates learning, but because it can provide recognition, guidance, interpersonal security, and opportunities to interpret personal experiences and goals. These interpersonal experiences may, in turn, be associated with how students understand the purpose, coherence, and significance of their lives. Presence of meaning is therefore conceptualized as a more proximal psychological resource that may connect course-contextual supportive experiences with broader evaluations of life. Recent evidence among Chinese college students indicates that social support is positively associated with meaning in life (Lyu, 2024). More broadly, supportive interpersonal relationships may provide students with recognition, guidance, emotional security, and opportunities for reflection, all of which may be relevant to meaning-related psychological functioning. These considerations provide a basis for examining whether support perceived specifically from teachers is similarly associated with students’ presence of meaning.

This association may be especially relevant within IPE because its value-oriented content may invite students to reflect on personal goals, social roles, responsibility, and the broader significance of individual experience (Wang, 2026; Zhao and Yang, 2026). Within such a context, supportive teacher–student interactions may be associated not only with course learning but also with opportunities for students to connect abstract course themes with their lived experiences, clarify personally meaningful goals, and reflect on their place within broader social relationships. This interpretation is theoretically plausible but was not directly tested in the present study. Recent research on Chinese university ideological and political theory courses has identified teacher support and influence as a meaningful component of students’ educational experiences (Wang, 2026). Although direct evidence for a specific IPE teacher-support–meaning association remains limited, the available literature provides a plausible theoretical and empirical basis for expecting students who perceive greater support from IPE teachers to report a stronger presence of meaning in life.

*H1*. Perceived teacher support in ideological and political education is positively associated with presence of meaning in life.

### Perceived teacher support in ideological and political education and life satisfaction

Life satisfaction refers to individuals’ global cognitive evaluation of the quality of their lives and represents a central component of subjective well-being (Diener et al., 1985). Because life satisfaction is a broad evaluation rather than a course-specific outcome, any association with perceived teacher support should not be assumed to arise simply from instructional effectiveness. Instead, supportive teacher–student relationships may be relevant insofar as they contribute to students’ broader interpersonal and psychological experiences, including feeling recognized, respected, guided, and supported within the university environment. In this sense, course-contextual teacher support may correspond with global life evaluation when students interpret supportive educational relationships as part of their broader experience of university life.

Recent evidence from Chinese higher education supports the broader psychological relevance of supportive teacher–student relationships. Liu and Zhao (2026), for example, found that teacher emotional support was associated with psychological well-being through need satisfaction and autonomous motivation among Chinese undergraduates. Although psychological well-being is conceptually distinct from life satisfaction, this finding indicates that teacher support may correspond with outcomes extending beyond immediate academic performance. IPE may provide a particularly relevant context because it combines interpersonal interaction with value-oriented content concerning personal development and relationships between individuals and society. The present study therefore examines whether students who perceive greater instrumental, emotional, informational, and appraisal support from IPE teachers also report greater life satisfaction.

*H2*. Perceived teacher support in ideological and political education is positively associated with life satisfaction.

### Presence of meaning in life and life satisfaction

Presence of meaning in life reflects the extent to which individuals already experience their lives as meaningful and purposeful rather than the extent to which they are actively searching for meaning (Steger et al., 2006). A stronger presence of meaning may provide an organizing psychological framework through which individuals interpret experiences, connect current activities with valued goals, and evaluate their lives as coherent and worthwhile. This provides a direct psychological basis for expecting presence of meaning to be positively associated with life satisfaction.

Recent evidence strongly supports this relationship. Zhang et al. (2025), using a sample of 909 Chinese young adults from four public universities, found that presence of meaning was positively associated with life satisfaction even after accounting for selected background variables. In addition, a 2025 systematic review of 22 empirical studies involving 7,732 university students concluded that stronger meaning in life was consistently associated with greater life satisfaction, resilience, and psychological well-being (Ortega-Gómez et al., 2025). These findings provide both culturally relevant and broader university-level support for the proposed association.

*H3*. Presence of meaning in life is positively associated with life satisfaction.

### The indirect association through presence of meaning in life

Taken together, the preceding evidence suggests that presence of meaning may statistically link supportive teacher experiences with life satisfaction. Within the proposed model, perceived teacher support represents a contextual interpersonal resource embedded in the educational environment, whereas presence of meaning represents a more proximal psychological resource concerning how students understand the significance, direction, and coherence of their lives. Recent research supports the component associations. Social support has been associated with meaning in life among Chinese college students (Lyu, 2024), while teacher support has been linked with positive psychological functioning in Chinese higher education (Liu and Zhao, 2026). Together, these findings suggest that supportive interpersonal resources may be relevant to both meaning-related functioning and broader well-being, although the specific indirect association proposed in the present study has not been directly established.

What remains unclear is whether presence of meaning statistically links perceived teacher support with life satisfaction specifically within a value-oriented university educational context such as IPE. Existing IPE research has begun to connect students’ course experiences with psychological capital and subjective well-being (Zhao and Yang, 2026), and teacher support has been identified as a meaningful component of students’ experiences in ideological and political theory courses (Wang, 2026). However, these strands of research have not directly examined whether meaning in life accounts for part of the statistical association between perceived teacher support and life satisfaction. The proposed ordering therefore represents a theoretically specified pattern of association rather than an established temporal or causal mechanism.

*H4*. Within the theoretically specified model, perceived teacher support in ideological and political education is indirectly associated with life satisfaction through presence of meaning in life.

### Conceptual model

Taken together, H1–H4 form the proposed conceptual model shown in Figure 1. The model specifies direct associations of perceived teacher support in ideological and political education with presence of meaning in life and life satisfaction, an association between presence of meaning in life and life satisfaction, and an indirect association between perceived teacher support and life satisfaction through presence of meaning in life.

**Figure 1:**
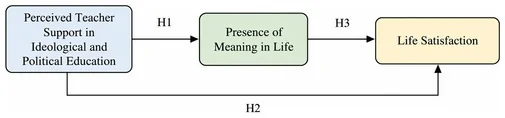
Conceptual framework of the proposed research model. Solid arrows denote the hypothesized associations tested in the structural model. The indirect association of perceived teacher support in ideological and political education with life satisfaction through presence of meaning in life (H4) is represented by the combined H1 and H3 paths. Because the variables were assessed concurrently, the arrows reflect the hypothesized model specification rather than established temporal ordering.

## Method

### Participants and procedure

This study employed a cross-sectional quantitative survey design to examine the associations among perceived teacher support in ideological and political education, presence of meaning in life, and life satisfaction among Chinese university students. Participants were 365 undergraduate students recruited from three higher education institutions in Guangxi, China, including one comprehensive university, one arts-focused university, and one vocational university. Eligible participants were full-time undergraduate students in Years 1 through 4 who had completed or were currently taking university ideological and political theory courses. Course-level, class-level, and instructor-level identifiers were not collected, so clustering and variation in instructional conditions could not be examined. Data were collected between February 19 and April 28, 2026, using Wenjuanxing (Questionnaire Star). Participants were recruited through convenience sampling using classroom-based access, and with the cooperation of course instructors, students completed the electronic questionnaire during scheduled class sessions. Before participation, students received an information statement explaining the study purpose, the voluntary nature of participation, confidentiality protections, and their right to discontinue the questionnaire at any point before submission without penalty. Electronic informed consent was obtained through an explicit consent item presented before the questionnaire. Students indicated consent by confirming that they had read the study information, understood the study, and voluntarily agreed to participate. Students were informed that participation or non-participation would not affect their course grades, academic evaluation, or relationship with their instructors and that individual responses would not be accessible to course instructors. No monetary compensation, course credit, gifts, lotteries, or other participation incentives were provided. The questionnaire was anonymous and did not collect personally identifiable information. Because individual responses could not be linked to identifiable participants after anonymous submission, post-submission withdrawal of individual responses was not possible.

A total of 365 students completed the survey. All substantive questionnaire items were configured as required-response fields in Wenjuanxing, which prevented submission when an item was left unanswered and therefore resulted in complete item-level data. All submitted responses met the eligibility criteria, and all 365 cases were retained for analysis. No missing-data imputation was required. No formal attention-check items were embedded in the questionnaire, and no additional *post hoc* screening based on completion time, duplicate-response detection, straight-lining, or other response-pattern criteria was applied. The sample included 161 students (44.1%) from the comprehensive university, 78 (21.4%) from the arts-focused university, and 126 (34.5%) from the vocational university; 164 participants (44.9%) were male and 201 (55.1%) were female. Students from all four undergraduate years and multiple major categories were represented. Detailed sample characteristics are presented in Table 1. Exact participant age was not collected; undergraduate year was used to characterize students’ stage of study.

**Table 1:** Characteristics of the study sample (*N* = 365).

Characteristic	Category	*n*	%
School type	Comprehensive university	161	44.1
	Arts-focused university	78	21.4
	Vocational university	126	34.5
Gender	Male	164	44.9
	Female	201	55.1
Grade	Freshman	109	29.9
	Sophomore	110	30.1
	Junior	79	21.6
	Senior	67	18.4
Major category	Arts	106	29.0
	Humanities/Social Sciences	100	27.4
	Science/Engineering	130	35.6
	Other	29	7.9

### Measures

#### Perceived teacher support in ideological and political education

Perceived teacher support in ideological and political education was assessed using a contextualized version of the Perceived Teacher Support Scale (PTSS; Wu et al., 2024). The original PTSS comprises 25 items assessing four dimensions of perceived teacher support: instrumental support, emotional support, informational support, and appraisal support. In the present study, the items were contextualized to university ideological and political theory courses by referring to the teacher as an IPE course teacher. Participants were instructed to reflect on their actual learning experiences in university IPE courses when responding. The substantive content of the 25 items, the four-dimensional structure, and the original six-point response format were retained, and no items were added or removed. The adapted measure was intended to assess students’ perceived teacher support within the IPE course context rather than overall instructional quality. The questionnaire referred generally to ‘the ideological and political theory course teacher’ and did not require students with experience in multiple IPE courses to identify a single current or most recent instructor. The resulting scores were therefore interpreted as course-contextual perceptions of IPE teacher support.

A sample item was, ‘My ideological and political theory course teacher takes the time to help me when I need help with my learning.’ Participants responded to the items using a six-point Likert-type scale ranging from 1 (strongly disagree) to 6 (strongly agree), with higher scores indicating greater perceived teacher support. Because the PTSS was originally developed with Chinese secondary-school students and was applied here to a university IPE context, the contextualized version was reviewed by two experts in educational psychology and higher education to assess the clarity, contextual appropriateness, and relevance of the adapted wording. A pilot test was then conducted with 30 university students to examine item comprehensibility and questionnaire administration. No substantive problems were identified, and no item modifications were required following the pilot test. The factorial structure and psychometric properties of the adapted PTSS were subsequently re-evaluated in the main study sample before structural analyses were conducted.

#### Presence of meaning in life

Presence of meaning in life was assessed using the five-item Presence of Meaning subscale of the Meaning in Life Questionnaire (MLQ; Steger et al., 2006). Because the questionnaire was administered in Chinese, the original English items were translated into Chinese before data collection. The translated items were reviewed by the research team for semantic clarity and consistency with the original item meanings. No independent back-translation procedure was conducted. The subscale assesses the extent to which individuals perceive their lives as meaningful, purposeful, and coherent. The Search for Meaning subscale was not included because the present study focused specifically on students’ perceived presence of meaning rather than their active search for meaning.

A sample item was, “I understand my life’s meaning.” Items were rated on a seven-point scale ranging from 1 (absolutely untrue) to 7 (absolutely true). One negatively worded item was reverse scored in accordance with the original scoring procedure. Higher scores indicated a stronger presence of meaning in life.

#### Life satisfaction

Life satisfaction was assessed using the five-item Satisfaction With Life Scale (SWLS; Diener et al., 1985), which measures individuals’ global cognitive evaluation of satisfaction with their lives. Because the questionnaire was administered in Chinese, the original English items were translated into Chinese before data collection. The translated items were reviewed by the research team for semantic clarity and consistency with the original item meanings before inclusion in the questionnaire. No independent back-translation procedure was conducted. A sample item was, “In most ways, my life is close to my ideal.” Items were rated on a seven-point scale ranging from 1 (strongly disagree) to 7 (strongly agree), with higher scores indicating greater life satisfaction.

### Data analysis

Data screening, descriptive statistics, and preliminary analyses were conducted using SPSS, whereas confirmatory factor analysis (CFA) and structural equation modeling (SEM) were performed in AMOS using maximum likelihood (ML) estimation. Means, standard deviations, skewness, kurtosis, and Pearson correlations were calculated for the focal study variables. Before testing the structural model, the psychometric properties of the measures were evaluated using Cronbach’s alpha, composite reliability (CR), standardized factor loadings, and average variance extracted (AVE). Discriminant validity among the four first-order teacher-support dimensions was additionally examined using the heterotrait–monotrait ratio of correlations (HTMT). Because the PTSS comprises four theoretically distinct dimensions, three alternative representations of teacher support were compared: a four-correlated-first-order-factor model, a second-order model in which the four support dimensions loaded on an overarching perceived teacher-support factor, and a single-factor model in which all PTSS items loaded directly on one factor. The broader measurement model was then evaluated with the four teacher-support dimensions, presence of meaning in life, and life satisfaction represented as six correlated first-order factors. If the second-order PTSS model demonstrated acceptable fit and provided a theoretically defensible representation of the four related support dimensions, the second-order perceived teacher-support factor was used as the predictor in the structural model. Model selection was therefore based on both empirical adequacy and theoretical alignment with the hypothesized structural model rather than on superior comparative fit alone. Model fit was evaluated using the chi-square statistic, comparative fit index (CFI), Tucker–Lewis index (TLI), root mean square error of approximation (RMSEA), and standardized root mean square residual (SRMR), with interpretation based on the overall pattern of fit indices and conventional benchmarks. Potential common method variance was considered procedurally through anonymity, confidentiality, voluntary participation, and separation of the focal constructs into different questionnaire sections, and was examined statistically using Harman’s single-factor test and comparison of a single-factor CFA model with the hypothesized multidimensional measurement model. These statistical procedures were treated as limited diagnostics rather than as tests capable of ruling out common method variance. The primary SEM was estimated without demographic or educational covariates. To examine the robustness of the focal associations, a supplementary sensitivity analysis was conducted using scale-score regression models controlling for gender, grade level, academic major, and institution type. For the supplementary analysis, perceived teacher support was represented by the mean of the 25 PTSS items, presence of meaning by the mean of its five items after reverse scoring the negatively worded item, and life satisfaction by the mean of the five SWLS items. Direct, indirect, and total structural associations were evaluated using 5,000 bias-corrected bootstrap resamples and were considered statistically supported when the corresponding 95% bootstrap confidence interval did not include zero. Bias-corrected confidence intervals for the focal direct and indirect associations in the supplementary sensitivity analysis were likewise estimated using 5,000 bootstrap resamples.

## Results

### Preliminary analyses and common method bias

The distributions of the focal variables showed no substantial departures from univariate normality. Skewness values ranged from −0.317 to −0.280, and kurtosis values ranged from −0.593 to −0.462. Potential common method bias was examined using Harman’s single-factor test and a single-factor confirmatory factor analysis (CFA). The first unrotated factor accounted for 30.5% of the total variance. In addition, the all-items single-factor CFA model showed poor fit, *χ*^2^(560) = 3347.07, CFI = 0.558, TLI = 0.531, RMSEA = 0.117, and SRMR = 0.123. As shown in Table 2, the single-factor model fitted substantially worse than the hypothesized multidimensional measurement model, indicating that a single common factor did not adequately account for the covariance structure of the study measures. However, these diagnostic results do not rule out other forms of shared method variance.

**Table 2:** Fit indices for the measurement models.

Model	*χ* ^2^	df	CFI	TLI	RMSEA	SRMR
PTSS four-correlated-factor model	409.25	269	0.970	0.966	0.038	0.043
PTSS higher-order model	421.87	271	0.967	0.964	0.039	0.046
PTSS single-factor model	1568.61	275	0.720	0.694	0.114	0.096
Hypothesized six-factor measurement model	790.00	545	0.961	0.958	0.035	0.049
All-items single-factor model	3347.07	560	0.558	0.531	0.117	0.123

### Measurement model, reliability, and validity

The measurement properties of the study constructs were evaluated before testing the structural model. As shown in Table 2, the correlated four-factor PTSS model demonstrated good fit, *χ*^2^(269) = 409.25, CFI = 0.970, TLI = 0.966, RMSEA = 0.038, and SRMR = 0.043. The higher-order PTSS model also showed good fit, *χ*^2^(271) = 421.87, CFI = 0.967, TLI = 0.964, RMSEA = 0.039, and SRMR = 0.046. The standardized loadings of the four first-order dimensions on the second-order perceived teacher-support factor were 0.777 for instrumental support, 0.859 for emotional support, 0.814 for informational support, and 0.665 for appraisal support. In contrast, the single-factor PTSS model fitted the data poorly, *χ*^2^(275) = 1568.61, CFI = 0.720, TLI = 0.694, RMSEA = 0.114, and SRMR = 0.096. These results supported the four-dimensional structure of perceived teacher support. Although the correlated four-factor model showed slightly better fit, the second-order PTSS model also demonstrated good fit. The second-order representation was selected on theoretical and parsimony grounds, rather than because it provided superior fit to the correlated four-factor model, because the hypothesized structural model treated perceived teacher support as an overarching construct represented by its four dimensions. It was therefore used in the subsequent structural analysis. Separately, the overall measurement model in which instrumental, emotional, informational, and appraisal support, presence of meaning in life, and life satisfaction were represented as six correlated first-order factors also demonstrated good fit, *χ*^2^(545) = 790.00, CFI = 0.961, TLI = 0.958, RMSEA = 0.035, and SRMR = 0.049.

Reliability and convergent-validity statistics are presented in Table 3. Standardized factor loadings ranged from 0.485 to 0.846. Cronbach’s alpha coefficients ranged from 0.834 to 0.903 across the four teacher-support dimensions and were 0.840 for presence of meaning in life and 0.862 for life satisfaction. Composite reliability values ranged from 0.834 to 0.903 for the teacher-support dimensions and were 0.845 for presence of meaning in life and 0.864 for life satisfaction. AVE values ranged from 0.502 to 0.573 across the study constructs. Taken together, these results supported acceptable internal consistency and convergent validity.

**Table 3:** Reliability and convergent validity of the study constructs.

Construct	Cronbach’s *α*	CR	AVE	Standardized loading range
Instrumental support	0.890	0.892	0.537	0.651–0.806
Emotional support	0.860	0.861	0.509	0.615–0.774
Informational support	0.903	0.903	0.573	0.672–0.846
Appraisal support	0.834	0.834	0.502	0.688–0.724
Presence of meaning in life	0.840	0.845	0.529	0.485–0.828
Life satisfaction	0.862	0.864	0.560	0.687–0.819

CR, composite reliability; AVE, average variance extracted.

Discriminant validity was examined using the heterotrait–monotrait ratio of correlations (HTMT). HTMT values among the four first-order teacher-support dimensions ranged from 0.511 to 0.689, remaining below commonly used thresholds for discriminant validity. These results supported the empirical distinctiveness of the four teacher-support dimensions.

### Descriptive statistics and correlations

Descriptive statistics and zero-order correlations among the focal variables are reported in Table 4. Perceived teacher support had a mean of 4.05 (SD = 0.90) on the six-point response scale. Presence of meaning in life had a mean of 4.45 (SD = 1.32), and life satisfaction had a mean of 4.65 (SD = 1.35), both measured on seven-point scales.

**Table 4:** Descriptive statistics and correlations among the study variables.

Variable	*M*	SD	Skewness	Kurtosis	1	2	3
1. Perceived teacher support	4.048	0.904	−0.280	−0.462	—		
2. Presence of meaning in life	4.447	1.319	−0.317	−0.476	0.240***	—	
3. Life satisfaction	4.650	1.352	−0.287	−0.593	0.282***	0.395***	—

***p < 0.001.

As shown in Table 4, perceived teacher support was positively correlated with presence of meaning in life, *r* = 0.240, *p* < 0.001, and life satisfaction, *r* = 0.282, *p* < 0.001. Presence of meaning in life was also positively correlated with life satisfaction, *r* = 0.395, *p* < 0.001. Thus, the directions of the zero-order associations were consistent with H1–H3.

### Structural model and hypothesis testing

The hypothesized structural model demonstrated good fit to the data, *χ*^2^(553) = 810.93, CFI = 0.959, TLI = 0.956, RMSEA = 0.036, and SRMR = 0.053. Standardized path coefficients and bootstrap results are presented in Table 5, and the standardized structural model is illustrated in Figure 2.

**Table 5:** Standardized structural associations and bootstrap results.

Hypothesis	Association	*β*	Bootstrap SE	95% BC bootstrap CI	Statistical support
H1	Teacher support → Presence of meaning	0.305	0.063	[0.181, 0.423]	Supported
H2	Teacher support → Life satisfaction	0.191	0.059	[0.076, 0.306]	Supported
H3	Presence of meaning → Life satisfaction	0.421	0.060	[0.299, 0.534]	Supported
H4	Teacher support → Presence of meaning → Life satisfaction	0.128	0.032	[0.073, 0.199]	Supported
–	Total association: Teacher support → Life satisfaction	0.319	0.056	[0.204, 0.424]	–

β = standardized estimate; SE = standard error; BC = bias-corrected; CI = confidence interval. Confidence intervals were based on 5,000 bootstrap resamples.

**Figure 2:**
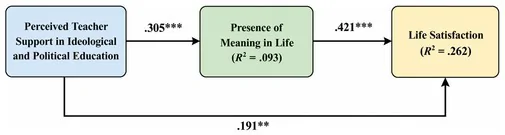
Standardized structural model results. Values shown on the paths are standardized coefficients. *R*^2^ values indicate explained variance. *p < 0.05, **p < 0.01, ***p < 0.001. The indirect association between perceived teacher support in ideological and political education and life satisfaction through presence of meaning in life was *β* = 0.128, 95% bias-corrected bootstrap CI [0.073, 0.199], based on 5,000 bootstrap resamples.

Perceived teacher support in ideological and political education was positively associated with presence of meaning in life, *β* = 0.305, Bootstrap SE = 0.063, 95% bias-corrected bootstrap CI [0.181, 0.423], providing statistical support for H1. Perceived teacher support also showed a positive direct association with life satisfaction after accounting for presence of meaning in life, *β* = 0.191, Bootstrap SE = 0.059, 95% BC CI [0.076, 0.306], providing statistical support for H2. Presence of meaning in life was positively associated with life satisfaction, *β* = 0.421, Bootstrap SE = 0.060, 95% BC CI [0.299, 0.534], providing statistical support for H3. As shown in Figure 2, the model explained 9.3% of the variance in presence of meaning in life and 26.2% of the variance in life satisfaction.

The 5,000-resample bootstrap analysis further indicated a positive indirect association between perceived teacher support and life satisfaction through presence of meaning in life, *β* = 0.128, Bootstrap SE = 0.032, 95% BC CI [0.073, 0.199]. Because the confidence interval did not include zero, the hypothesized indirect association specified in H4 received statistical support. The total association between perceived teacher support and life satisfaction was also positive, β = 0.319, Bootstrap SE = 0.056, 95% BC CI [0.204, 0.424]. As summarized in Table 5, the observed statistical associations were consistent with all four hypotheses.

### Sensitivity analysis with covariate adjustment

As a supplementary sensitivity analysis, the focal associations were re-examined using scale-score regression models controlling for gender, grade level, academic major, and institution type. The substantive pattern of focal associations remained consistent with the primary analysis. Perceived teacher support remained positively associated with presence of meaning in life (*β* = 0.243, 95% BC bootstrap CI [0.136, 0.348]) and life satisfaction (*β* = 0.200, 95% BC bootstrap CI [0.102, 0.297]), while presence of meaning in life remained positively associated with life satisfaction (*β* = 0.341, 95% BC bootstrap CI [0.243, 0.444]). The adjusted indirect association between perceived teacher support and life satisfaction through presence of meaning in life also remained positive and statistically supported (*β* = 0.083, 95% BC bootstrap CI [0.045, 0.137]). These results indicate that the substantive pattern of the focal associations was robust to adjustment for the measured demographic and educational characteristics.

## Discussion

The present study examined the direct and indirect associations among students’ perceived teacher support in ideological and political education, presence of meaning in life, and life satisfaction. Perceived teacher support was positively associated with both presence of meaning and life satisfaction, and presence of meaning was positively associated with life satisfaction. A significant indirect association was also observed through presence of meaning within the specified model. These findings concern students’ course-contextual perceptions of support within IPE rather than support attributable to a single identified instructor or independently observed teacher behavior. Accordingly, the results should not be interpreted as evidence that objectively measured supportive teaching behavior by a specific instructor produced the observed psychological outcomes.

### Perceived teacher support in ideological and political education and presence of meaning in life

Perceived teacher support in ideological and political education was positively associated with presence of meaning in life. This finding is consistent with evidence linking social support with meaning in life among Chinese college students (Lyu, 2024) and extends this broader interpersonal-support literature by focusing specifically on students’ course-contextual perceptions of teacher support within university IPE. In this value-oriented context, students engage with content related to personal development, social responsibility, and relationships between individuals and society. One theoretical interpretation is that perceptions of instrumental, emotional, informational, and appraisal support may be compatible with greater reflection, goal clarification, and interpretation of personal experiences. These processes were not directly measured in the present study and should therefore be regarded as plausible theoretical explanations rather than empirically established mechanisms. At the same time, perceived teacher support explained a relatively modest proportion of the variance in presence of meaning (*R*^2^ = 0.093), suggesting that teacher support represents only one of multiple resources associated with students’ meaning-related functioning. Family relationships, peer support, personal values, identity development, and broader life experiences may account for additional variation in students’ presence of meaning. However, the observed association does not establish that supportive teaching increases students’ meaning in life, and reverse or reciprocal relationships remain plausible.

### Perceived teacher support in ideological and political education and life satisfaction

Perceived teacher support was also positively associated with life satisfaction after presence of meaning was included in the model. This finding is broadly consistent with evidence that supportive teacher–student relationships remain psychologically relevant in higher education. Liu and Zhao (2026), for example, reported that teacher emotional support was associated with psychological well-being among Chinese university students through psychological need satisfaction and autonomous motivation. Although psychological well-being and life satisfaction are conceptually distinct, both findings suggest that supportive teacher experiences are related to outcomes beyond immediate academic performance. The current study adds context-specific evidence from university IPE and complements recent work linking IPE with psychological nurturing, psychological capital, and subjective well-being (Xue et al., 2024; Zhao and Yang, 2026). Nevertheless, life satisfaction is shaped by multiple personal and contextual factors, and the present association should not be interpreted as evidence that increasing teacher support will necessarily increase life satisfaction.

### Presence of meaning in life, life satisfaction, and the indirect association

Presence of meaning showed a comparatively strong association with life satisfaction. This is consistent with recent evidence among Chinese young adults and university students showing that stronger presence of meaning is associated with greater life satisfaction and psychological well-being (Zhang et al., 2025; Ortega-Gómez et al., 2025). Students who perceive their lives as purposeful, coherent, and significant may also report greater organization of their experiences around valued goals and more favorable global evaluations of life.

The significant indirect association through presence of meaning represents a statistical feature of the theoretically specified model rather than evidence of a temporal mediation process. Specifically, part of the covariance between perceived teacher support and life satisfaction was statistically represented through presence of meaning in life. Conceptually, the proposed ordering treats perceived teacher support as a contextual interpersonal resource and presence of meaning as a more proximal psychological resource. However, because all constructs were measured concurrently, the data do not establish that perceived teacher support temporally preceded presence of meaning or that presence of meaning subsequently preceded life satisfaction. The indirect association should therefore be interpreted as a model-dependent statistical decomposition rather than as evidence of causal mediation. Reverse, reciprocal, and alternative directional explanations remain plausible.

### Theoretical implications

The theoretical contribution of the study lies less in proposing that teacher support, meaning, and well-being are universally related than in specifying how these constructs may be connected across different levels of students’ educational and psychological experience. Perceived teacher support is course-contextual and relational, whereas presence of meaning concerns students’ broader sense of purpose, coherence, and significance, and life satisfaction reflects a still broader evaluation of life as a whole. Examining these constructs together therefore addresses whether a relatively proximal educational relationship corresponds with broader psychological functioning through a meaning-related resource. This question is particularly relevant in IPE because the curriculum explicitly engages value-oriented themes concerning personal development, social responsibility, and relationships between individuals and society. The findings extend teacher-support research into a value-oriented university IPE setting in which course content may invite reflection on personal development, social responsibility, and meaning-related concerns. However, because the study did not include a non-IPE comparison group and did not directly assess students’ engagement with value-oriented course content, the present data cannot determine whether the observed associations are specific to IPE or instead reflect broader teacher-support processes that may operate across university courses. Accordingly, the present theoretical contribution should be interpreted as context-specific evidence from Chinese university IPE rather than as evidence that the proposed association pattern generalizes across educational or cultural contexts.

Perceived teacher support should also be distinguished from broader teaching quality. Some PTSS items involving guidance, feedback, learning resources, and problem solving may overlap conceptually with instructional responsiveness. Future research should therefore assess teacher support and teaching quality as distinct constructs to examine their unique associations with meaning in life and life satisfaction.

### Practical implications

The findings identify perceived teacher–student support as one interpersonal correlate that may warrant attention alongside curriculum-focused approaches in university IPE. Given the observational design and the relatively modest proportion of variance in presence of meaning explained by teacher support, the findings do not establish that increasing teacher support would produce corresponding increases in meaning in life or life satisfaction. Teacher support should therefore be viewed as one of several potentially relevant interpersonal and contextual resources rather than as a primary determinant of these outcomes. Moreover, the present findings should not be interpreted as isolating perceived teacher support from broader teaching quality. In particular, students’ perceptions of instrumental, informational, and appraisal support may partly reflect instructional responsiveness, the availability of learning assistance, and the quality of feedback provided within the course. Accordingly, supportive interaction and instructional quality should be regarded as potentially overlapping aspects of students’ course experiences rather than as fully separable components of the educational experience. The present study also does not estimate the incremental contribution of perceived IPE teacher support beyond family support, peer support, broader social support, personal characteristics, or other educational experiences. Thus, the observed associations should not be interpreted as outcomes attributable uniquely or solely to teacher support within IPE. At the conceptual level, students’ course-contextual perceptions of instrumental support may reflect experiences of receiving assistance with learning difficulties and access to relevant resources; perceived emotional support may reflect respectful, responsive, and encouraging interactions; perceived informational support may reflect clear guidance that assists interpretation of course content; and perceived appraisal support may reflect constructive feedback concerning strengths and areas for development. In the specific context of IPE, supportive interaction may also include opportunities for students to connect abstract value-oriented course themes with their lived experiences, personal goals, social roles, and broader questions of responsibility and purpose. Such practices are conceptually consistent with a more supportive interpersonal dimension of IPE, but the present findings do not establish that adopting them would increase students’ meaning in life or life satisfaction.

### Limitations and future research

Several limitations qualify the interpretation of the findings. First, the cross-sectional design does not establish temporal ordering or causality, and reverse or reciprocal relationships remain plausible. Students with a stronger pre-existing sense of meaning or greater life satisfaction may, for example, perceive their teachers and educational experiences more positively. Although supplementary analyses indicated that the focal associations remained robust after adjustment for gender, grade level, academic major, and institution type, unmeasured third variables may still contribute to the observed associations. The study did not assess several potentially relevant factors, including family and peer support, socioeconomic background, academic stress and performance, personality and affective characteristics, and students’ prior attitudes toward or engagement with IPE. Accordingly, the directional paths in the structural model should be interpreted as theoretically specified associations rather than empirically established temporal processes.

Second, all focal constructs were assessed using student self-report within the same survey administration. Shared response tendencies, consistency motives, or general positive response styles may therefore have contributed to the observed associations. Although Harman’s single-factor test and the poor fit of the all-items single-factor CFA indicated that a single common factor did not adequately account for the covariance structure, these procedures cannot rule out common method variance. Perceived teacher support also reflects students’ subjective appraisals rather than independently observed teacher behavior. In addition, the PTSS was originally developed for secondary-school students and was contextually adapted to university IPE. Although the contextualized wording was reviewed by two experts, pilot tested with 30 university students, and showed acceptable factorial, reliability, convergent-validity, and discriminant-validity results in the present sample, these procedures do not constitute comprehensive validation of the instrument for university IPE settings. Future studies should incorporate multiple sources of information, such as teacher reports, classroom observations, and instructional-quality measures, and further examine the content validity and psychometric performance of the adapted PTSS in university IPE settings.

Third, the sample was recruited through classroom-based convenience sampling from three higher education institutions in Guangxi, China, which limits generalizability to other regions and university contexts. Class- and instructor-level identifiers were not collected, and students were not required to rate a single standardized focal instructor; consequently, potential clustering and variation in instructional conditions could not be examined. Future research should distinguish between generalizability within Chinese higher education and transferability across educational and cultural contexts. Replication across regions and institutional types within China would help assess the robustness of the observed association pattern, but would not establish whether these relationships are specific to IPE or reflect broader teacher-support processes. Direct comparisons between IPE and non-IPE university courses are therefore needed. Cross-context studies could also examine functionally comparable value-oriented educational settings outside Chinese IPE without assuming direct equivalence across educational systems or cultures. Before comparing structural relations across educational or cultural groups, future research should establish measurement invariance of the relevant measures. Studies should also retain class- and instructor-level identifiers to permit cluster-adjusted or multilevel analyses where warranted, and longitudinal designs could examine whether the observed associations persist beyond the IPE course context.

## Conclusion

This study examined the associations among perceived teacher support in ideological and political education, presence of meaning in life, and life satisfaction among Chinese university students. Perceived teacher support was positively associated with both presence of meaning and life satisfaction, and presence of meaning was positively associated with life satisfaction. A significant indirect association further indicated that presence of meaning was statistically relevant to the association between perceived teacher support and life satisfaction within the specified model. The findings extend teacher-support research into the value-oriented context of university IPE by showing that students’ course-contextual perceptions of instrumental, emotional, informational, and appraisal support are associated with both meaning-related functioning and broader evaluations of life. They also highlight presence of meaning as a theoretically relevant psychological resource within the specified association pattern. Overall, the study contributes context-specific evidence that students’ perceptions of supportive teacher–student relationships within university IPE may represent one meaningful interpersonal correlate of psychological functioning. These findings should be interpreted as cross-sectional statistical associations rather than evidence of temporal mediation or causal effects.

## Data Availability

The raw data supporting the conclusions of this article will be made available by the authors, without undue reservation.
